# Association of Chondrolabral Lesions with Ultrasound-Guided Detection of Pathological Head–Neck Contour

**DOI:** 10.3390/diagnostics13213334

**Published:** 2023-10-29

**Authors:** Christian T. Schamberger, Christopher Tuffs, Arnold J. Suda, Tobias Grossner, Gerhard Schmidmaier, Stephan Stein

**Affiliations:** 1 Clinic for Trauma and Reconstructive Surgery, University Hospital Heidelberg, 69118 Heidelberg, Germany; 2 Department of General, Visceral and Transplantation Surgery, University Hospital Heidelberg, 69120 Heidelberg, Germany; 3 Department of Orthopaedics and Trauma Surgery, AUVA Trauma Center Salzburg, 5010 Salzburg, Austria

**Keywords:** femoroacetabular impingement, ultrasound, MRI, impingement

## Abstract

Objective: This study aimed to investigate whether the asphericity of the neck–head junction of the femur confirmed via ultrasound is associated with further pathology due to femoro-acetabular impingement (FAI). Methodology: After a clinical examination with positive FAI tests, an ultrasound examination of the hip was performed. In the case of asphericity, a quantitative ultrasound-assisted assessment of the hip was performed, followed by contrast-enhanced arthro-MRI with the question of cartilage or labral damage. Results and Conclusions: We included 51 patients with a mean age of 35.25. According to the examination algorithm, asphericity was present in all patients via ultrasonography. The average anterior alpha angle (AAA) determined in ultrasonography was 43.49°. The average AAA on the arthro-MRI was 44.19°. The mean anterior head neck offset (AHNO) in ultrasound was 5.27 mm, and in arthro-MRI, it was 5.36 mm. Arthro-MRI confirmed a bump in 47 patients and a talization disorder in 4 patients. In 49 patients, a labral lesion was found, with one being a re-rupture. Furthermore, in one patient, labral degeneration was identified. Cartilage damage to the hip joint was found in 25 patients. Two patients had neither labral nor cartilage damage in the arthro-MRI. In our study, sonographically confirmed asphericity of the head–neck junction was found in 49 cases, which was associated with further pathology and, according to the current doctrine, was attributable to the FAI and required surgical intervention. This study shows that the detection of a pathologic head and neck contour via ultrasound in combination with positive clinical signs, as present in FAI, is associated with chondrolabral lesions detected via arthro-MRI in 96.1% of cases.

## 1. Introduction

Femoroacetabular impingement syndrome (FAI) is a common pathology in the clinical setting of orthopedics [1,2,3]. Femoroacetabular impingement refers to the abnormal morphology of the hip joint characterized by pathological contact between the anterior upper femoral neck and the acetabulum [4]. It can be classified into two types: cam impingement, where there is a pathological transition of the femoral head and neck, and pincer impingement, where the femoral head is excessively covered by the acetabulum. Both configurations lead to pathological contact between the joint partners and cause various pathological changes in the hip joint attributed to FAI. In addition to typical clinical symptoms such as pain, FAI can cause damage to the articular cartilage and labrum, thereby increasing the risk of developing hip osteoarthritis [5,6]. Currently, diagnosis is performed via clinical examination, followed by plain radiographs (anteroposterior and axial views) and imaging techniques such as CT or MRI. Recent studies have shown that the changes in the head and neck can not only be visualized sonographically but also quantitatively measured, including the alpha angle and measurement of the anterior head–neck offset. The high comparability of the measurement results of MRI and ultrasound has also been confirmed in recent research [7]. Ultrasound examination offers several advantages as a cost-effective, reproducible, and widely available method in both outpatient and inpatient care in German-speaking countries. Its non-use of ionizing radiation is particularly noteworthy, setting it apart from conventional imaging methods and providing valid results [7]. Furthermore, ultrasound allows for the dynamic assessment of hip joint mobility, which can be crucial for the diagnosis and treatment of FAI. In addition to diagnostic benefits, real-time imaging with ultrasound allows for dynamic loading tests of the hip joint during movement, which can be valuable, especially in assessing impingement-related abnormalities [8,9]. Moreover, ultrasound is a non-invasive procedure, making it suitable for repeated examinations and follow-up assessments [1]. Based on an ultrasound-based evaluation of the hip joint and its advantages, early intervention and preventive measures may be enacted for patients at increased risk of FAI-related complications. In summary, ultrasound examination demonstrates great potential as a valuable tool for the diagnosis and monitoring of femoroacetabular impingement syndrome due to its cost-effectiveness, avoidance of ionizing radiation, and capability for dynamic imaging [1,9,10,11].

Further research and validation studies are needed to fully understand the extent of an ultrasound’s usefulness in FAI management and improving patient outcomes.

This study aimed to investigate whether the asphericity of the femoral head–neck junction assessed via ultrasound examination correlates with the pathological changes in the hip joint, such as labrum or cartilage damage resulting from FAI, which could reduce the need for further diagnostics, such as contrast-enhanced MRI and ionizing radiation-based tools. Additionally, the early identification of FAI and its complications could lead to initiating appropriate therapy sooner, thus avoiding long-term complications like hip osteoarthritis.

## 2. Methods

### 2.1. Study Design

This prospective clinical study was developed at the Center for Orthopedic and Trauma Surgery, University Hospital Mannheim. Ethical approval was obtained from the Ethics Committee of the Medical Faculty Mannheim, University Heidelberg (2020-840R). It was performed in accordance with the “Strengthening the Reporting of Observational Studies in Epidemiology (STROBE) Statement: guidelines for reporting observational studies” [12].

### 2.2. Patient Recruitment Inclusion and Exclusion Criteria

Patients were recruited for study participation between 1 January 2018 and 31 October 2020.

The inclusion criteria were the presence of an arthro-MRI of the hip in the femoral neck plane and a sonographic investigation of the identical joint in a modified anterior longitudinal view according to the DEGUM guidelines (German Society for Medical Ultrasound) using a linear ultrasound transducer with a variable frequency from 3.5 to 13 MHz (MyLab Six, ESAOTE Biomedica Germany, Genua, Italy).

The exclusion criteria were coxarthrosis diagnosed via MRI, femoral head necrosis, open epiphyseal plate, previously recorded pediatric diseases of the hip (such as M. Legg-Calvé-Perthes, epihysiolysis capitis femoris, etc.), fresh or consolidated fractures of the coxal femur or the acetabulum, total/semi hip arthroplasty, and technically insufficient results from MRI or ultrasound diagnostics for any reason.

### 2.3. Study Procedure, Materials, and Evaluation

Based upon the recommendation proposed by Schamberger et al., the hip joint was clinically examined by an attending physician for orthopedic and trauma surgery [1]. The clinical examination included a passive and active range of motion, the FABER test, the FADIR test, and the anterior impingement test [13,14]. The hip joints of patients presenting symptoms of femoroacetabular impingement were then examined via ultrasound using a device provided by the company ESAOTE^®^, type Mylab Six, using a linear transducer with variable frequencies between 3.5 and 13 MHz in the standard sections described in the DEGUM in 15° external rotation of the hip joint to evaluate the head–neck junction of the hip joint [15]. The femoral neck is positioned parallel to the upper screen margin, and the bone surface of the femoral neck must appear homogenous and hyperechoic [15]. For cam-evaluation, the transducer must be positioned anteriorly above the highest point of the femoral head [1,15,16]. Due to the acetabular edge, the bump cannot be seen during the internal rotation of the hip, and the evaluation of the head of the femur is impossible to extend to a full circle [1].

All examinations were conducted by experienced, DEGUM-certified investigators. In patients with signs of femoral asphericity, in the sense of pathological head–neck junction, quantification, as described by Schamberger et al., was conducted to measure the anterior alpha angle and the anterior head–neck offset via ultrasound examination [1]. Both evaluations via ultrasound examination and an MRI-based estimation of the anterior alpha angle and anterior head–neck offset were conducted by two independent investigators, both DEGUM-certified in musculoskeletal ultrasounds. In total, 24 measurements per joint were completed.

#### 2.3.1. Evaluation of Anterior Alpha Angle

**Figure 1:**
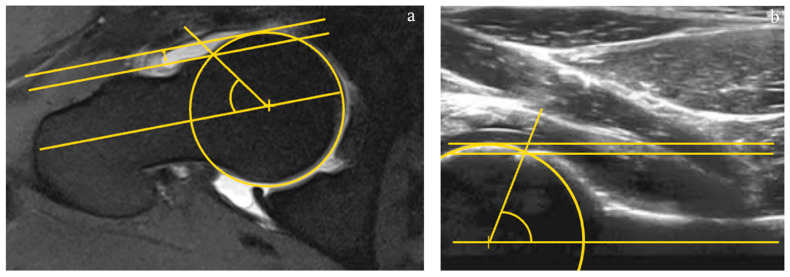
Evaluation of the anterior alpha angle and anterior head–neck offset in arthro-MRI in axial section (**a**) and in ultrasound in anterior longitudinal section according to DEGUM guidelines (**b**).

For ultrasound-assisted evaluations of the anterior alpha angle first, the center of the femoral head was identified using the circle tools of Weasis DICOM medical Viewer, 2020. The first arm of the angle was positioned as a line along the axis of the horizontally aligned femur. The second side of the angle was positioned as a line through the previously defined center of the head and the point where the shape of the femoral head changes into an aspherical shape, according to Noetzli et al. [17]. The same software and technique were used for the MR-tomographic determination of the alpha angle. The image showing the biggest plain head diameter with the largest cam deformation was used for the measurement (Figure 1a).

#### 2.3.2. Evaluation of the Anterior Head–Neck Offset

Just as for the evaluation of the alpha angle, in the sonographs, we first identified the femoral neck axis and the center of the femoral head. According to Eijer et al., a tangent to the highest expression of the femoral head and parallel to the neck axis was drawn using the same software mentioned above [18]. The femoral head segment was expanded to a full circle, and the spot where the femoral head leaves the sphericity was identified. Another parallel line to the femoral neck axis passing through this point was drawn. Then, the distance between these two lines was measured for the anterior head–neck offset [18,19,20]. The same software and technique were used for the MR-tomographic assessment of the anterior head–neck offset (Figure 1b).

#### 2.3.3. Arthro-MR-Tomographic Assessment

**Figure 2:**
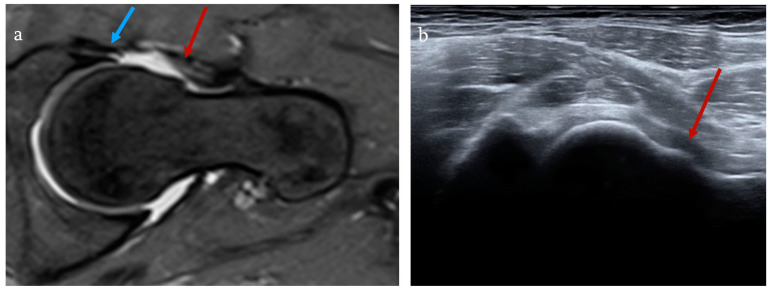
FAI with cam deformity (red arrow) and labral tear (blue arrow). Contrast-enhanced arthro-MRI (**a**) and ultrasound in anterior longitudinal section (**b**).

After finishing the ultrasound examination, a contrast-enhanced artho-MRI of the pathological hip joint was performed to detect lesions of the labrum and secure a diagnosis of femoroacetabular impingement. In all patients who were included in this study, an artho-MRI was completed using the parameters of 15 mL of intraarticular 2 mM gadolinium solution in the following sequences: axial FSE T1 Fat-Sat, coronary SE T1, coronary T2 Fat-Sat, sagittal FSE DP, axial three-dimensional (3D) DESS with radial reconstruction along the longitudinal axis of the femoral neck, or radial FSE DP image (2D) orientation being based upon the longitudinal femoral neck axis. Lesions of the labrum were diagnosed by both an orthopedic surgeon as well as a radiologist. Clinical examination and further diagnostics excluded other underlying pathologies explaining the presented symptoms (Figure 2).

#### 2.3.4. Statistical Analysis

Using the Student’s *t*-test, *p* < 0.05 was determined to be statistically significant. With *n* = 51, a statistical power of 0.9, and a level of significance of α = 0.05, the power analysis resulted in a correlation of r = 0.433 for significant results, according to Green et al. [21]. The statistical analyses of the means were conducted using SPSS version 27 (IBM SPSS Statistics for iOS, version 27.1, Armonk, NY, USA).

## 3. Results

### 3.1. Demography

**Scheme 1:**
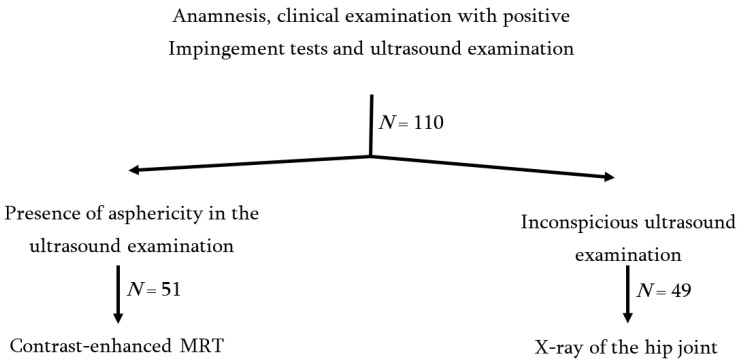
Diagnostical algorithm according to Schamberger et al. [1].

Out of 110 patients who underwent clinical examination with positive FAI-Tests, 51 patients were included based upon the previously mentioned inclusion and exclusion criteria (Scheme 1), and 49 patients were not included in this study. However, they underwent further examination according to the examination algorithm (Scheme 1). The mean age was 35.25 (15–65 a, SD 12.912), with 31 being female (60.8%) and 20 being male (39.2%). Each ultrasound examination and each MRI examination were measured three times by two independent examiners, so a total of 1224 measurements were performed on 51 hip joints. In all 110 patients, there were clinical signs of impingement in the hip joint. Following the algorithm of diagnostics, asphericity was present in 51 patients, while in 47 patients (92.2%), there was a bump with pathological reference values for AAA and AHNO. However, in eight patients (15.7%), insufficient femoral head–neck junction in the sense of a cam impingement with non-pathological reference values for AAA, AHNO, or a combination of both were present.

### 3.2. Anterior Alpha Angle

In total, 612 measurements were performed in 51 joints for the evaluation of the alpha angle in MRI and ultrasound-assisted evaluations. The mean alpha angle in MRI was 44.19° (range: 33.5° to 63.7°, SD 7.076), and the intra- and interobserver reliability was factual. The mean alpha angle in sonography was 43.5° (range: 32.6° to 62.4°, SD 7.078). The mean difference of alpha angles in MRI and sonography was 0.7° (SD 0.258) with a maximal deviation of 1.3°. The Bland–Altman plot shows a high correlation between sonographic and MRI measurements of the alpha angle. Pearson’s correlation was 0.999, which was statistically significant for both imaging methods. Regardless of the presence of cam deformity, the measurements in MRI and sonography showed comparable results. The presence of cam deformity did not influence the accuracy of both measurements. However, small alpha angles made the positioning of the second-angle side more difficult as a result of a physical ultrasound phenomenon [1] (Table 1 and Table 2).

### 3.3. Anterior Head–Neck Offset

In total, 612 measurements were performed in 51 hip joints for the evaluation of the anterior head–neck offset in MRI and ultrasound-guided estimations. In MRI, a mean head–neck offset of 5.36 mm (2.69–9.61 SD 1.48) was determined. The ultrasound-guided estimation of the anterior head–neck offset showed a mean of 5.26 mm (2.0–9.48 mm, SD 1.51). The mean difference between measurements in MRI and sonography was 0.097, with a maximum deviation of 1.6 mm (SD 0.218). Using SPSS Statistics (Vers. 27, IBM), Pearson’s correlation coefficient was 0.989, which is a strong correlation between both methods (*p* < 0.001). There were no outliers identified. The presence of a head–neck deformity did not influence the measurement’s accuracy. The Bland–Altman plot shows a high correlation between the sonographic and MRI measurements of the head–neck offset (Table 1 and Table 2).

### 3.4. Arthro-MR-Tomographic Assessment

Arthro-MRI showed a labral tear in 49 patients (96.1%), while in one of these patients (2.04%), a re-rupture of the labrum was diagnosed. In another patient, the degeneration of the acetabular labrum was detected. In total, 25 patients (49%) displayed cartilage damage in the hip joint. However, in two patients (3.9%), there was neither damage to the labrum nor cartilage, as detected via arthro-MRI, while labropathy was subsequently detected intraoperatively (Table 1 and Table 2).

## 4. Discussion

This study shows that the clinical manifestation of FAI, as well as the asphericity of the head–neck junction detected via ultrasound, is associated in 96.1% with lesions of the acetabular labrum and/or damage of the articular cartilage. In previous studies, most patients displaying a pathological head–neck junction also presented with existing or developing lesions of the chondrolabral region of the hip joint [5,22]. However, common clinical practice dictates the diagnosis of FAI via CT scan, exposing the patient to radiation, which is only then followed by MRI diagnostics [20,23].

The provided data estimated a mean alpha angle of 43.49° via ultrasound-guided examination and 44.19° via MRI imaging. These values are lower than those of previous studies, where the alpha angle was calculated using MRI images or radiographs, which raises the question of whether a cam deformity can be visualized earlier in ultrasounds than conventional procedures [24]. Despite discrepancies between the results with recommended guidelines of 50–60°, there was a high correlation between MRI and ultrasound-estimated alpha angle, as described in preceding studies [1,25]. Furthermore, almost all patients displayed a low-grade chondrolabral lesion in the following MRI of the hip joint. The threshold value for pathological alpha angle is currently defined as 60° [26]. Other authors define a critical angle as 55° or 50.5° [27,28]. After investigating 35 hip joints using X-ray images (45° Dunn view, 90° Dunn view, cross-table view) and MRI images, Saito et al. revealed that depending on the type of X-ray, there were differences between alpha angles, ranging from 46.8° to 59.7° [29]. Since the determination of the alpha angle is highly inconsistent, even with conventional diagnostic methods such as MRI, CT, and conventional X-rays, further studies should be conducted in the context of femoroacetabular impingement and the correlation of the threshold values of the alpha angle with the presence of bumps and lesions of the cartilage. Standard values assessed via ultrasound-guided examination need to be defined.

In this population, asphericity and associated pathologies were detected early, whereas in this population, no high-grade cartilage damage could be detected. Using highly standardized detectors and patient positions during the examinations, as defined in the DEGUM and previous studies, allows for the exact horizontal depiction of the femoral neck and sharp visualization of the corticalis [1,15,16], which eliminates variances in observed measurements compared to conventional X-ray imaging. This could be one of the reasons for the observed low alpha angle and subsequent diagnosis of FAI. During ultrasound-guided assessment of cam deformity, the bump is always displayed in its most extended shape, which can be difficult when using X-ray images. In addition to the static examination method, as conducted in this study, a dynamic investigative technique, as introduced by Billham et al. in 2021, could potentially become a diagnostic approach for directly representing a suspected labral injury in the future [8].

During this study, anatomical variants, such as coxa profunda, a retroversion of the articulated socket, or a high pelvic incidence, which could explain low alpha angles in combination with chondrolabral lesions, were not taken into consideration, limiting the results [30]. However, Clohisy et al. showed that damage to the labrum caused by pincer impingement is only present in 7.9% of patients in a collective of 1.130 observed hips [31]. Collins et al. do not recommend surgical therapy in asymptomatic patients with cam impingement since there is no evidence for improvement in the course of the disease [32]. Further complicating the matter, Heerey et al. detected cartilage and labral damage in football players with both asymptomatic and symptomatic cam deformities [22]. However, damage to the joint can also occur without symptoms of pain, and patients with unspecific symptoms are often unable to describe pain coming from the hip or groin. Patients from our collective were all symptomatic, with pain around the inguinal region as well as positive impingement tests, as previously described. Patients with functional disorders of the lumbar spine and sacroiliac joints were excluded. After a clinical examination was performed and the suspected diagnosis was FAI, the diagnosis was confirmed using a quantitative assessment via ultrasound examination measuring the anterior alpha angle and anterior head–neck offset.

In a multi-center study, Griffin et al. found that surgical treatment is superior to the conservative treatment of femoroacetabular cam impingement [33]. Considering this, diagnostic algorithms for FAI utilizing ultrasound diagnostics in symptomatic patients can detect early stages of chondrolabral damage, which, in turn, allows for surgical treatment without further diagnostics utilizing radiation, such as CT or conventional X-rays [1].

Besides the surgical treatment of femoroacetabular impingement (FAI), conservative therapeutic approaches are also discussed in the current literature. In a more recent publication in 2023, in which injections and physical therapy were combined, Ebert et al. were able to achieve a significant improvement in the clinical findings for 32% of their patient cohort in a 2-year follow-up, although no long-term results were described [34]. Despite the therapeutic successes presented in the previously mentioned study using conservative therapy for FAI, a meta-analysis from the year 2020 was already able to demonstrate that surgical treatment is significantly superior to non-operative therapy [35].

Previous studies showed a high sensitivity for MRI diagnostics for the detection of labral damage compared to intraoperative detection. In a study conducted by Sutter et al., MRI sensitivity was 85%, while Tian et al. reported a sensitivity of 90–95% [36,37]. We achieved a sensitivity of 96.1% and a specificity of 100% for the detection of chondrolabral damage, comparing a combination of clinical examination and ultrasound diagnostics for cam deformity versus contrast-enhanced MRI alone. Since patients were only included after confirmation of cam deformity via ultrasound examination, there is a high prevalence compared to previous studies for the presence of chondrolabral damage, e.g., cartilage damage, labral damage, or labropathy. Sensitivity for clinical tests regarding FAI or associated labral damage varies, depending on the examined literature, between 11 and 100%, while a specificity of 56% is achievable [38,39,40,41]. As previously described, specificity can be increased for clinical tests by excluding or quantitatively evaluating FAI using ultrasound diagnostics [1,24].

A limitation of our study is that only patients who had previously confirmed FAI were included. Still, we show that a combination of ultrasound and subsequent MRI imaging of the hip joint is sufficient to plan further surgical treatment. In all patients, no further X-ray diagnostics were necessary, which led to no decrease in diagnostic quality. Ultrasound diagnostics are superior compared to conventional radiological imaging, as they spare the patient from radiation exposure. Furthermore, it is a readily available, cheap, and widely spread method [1,15,16,42]. As a method to investigate the hip joint, it can also detect pathological changes in the surrounding soft tissue next to the assessment of FAI, which, in turn, requires no further diagnostics. In comparison to conventional X-ray diagnostics, which require an adequate positioning of the patient to determine the alpha angle and the anterior head–neck offset, images obtained using ultrasound diagnostics can be improved by repositioning the patient or probe without effort until an optimal image to estimate needed parameters is obtained [28]. In conventional cross-sectional imaging, the maximum length of the bump can lie between 2 and 4 mm thick sections [7]. This source of error is eliminated using ultrasound diagnostics since it is a dynamic method with live images [15]. Subcortical changes cannot be evaluated using ultrasound diagnostics. However, following the diagnostic algorithm, these become apparent in performed MRI diagnostics.

## 5. Conclusions

This study shows that the detection of a pathologic head and neck contour via ultrasound in combination with positive clinical signs, as present in FAI, is associated with chondrolabral lesions detected via arthro-MRI in 96.1% of patients. In contrast to previous studies in which the critical values for a pathological alpha angle were defined as 50–55°, chondrolabral damage was already present at a sonographically determined alpha angle of 44°. In our opinion, further X-ray diagnostics are not necessary for the evaluation of FAI since further relevant information cannot be collected.

Further studies should investigate correlations between ultrasound-diagnosed FAI and corresponding pathologies requiring surgical therapy. In the coming years, it can be expected that improvements in the quality and resolution of ultrasonography will lead to increasing importance in the diagnosis of FAI.

## Figures and Tables

**Table 1:** Quantitative evaluation of the hip joints in ultrasound and MRT.

	Head–Neck Offset (mm)	SD	Alpha Angle (°)	SD
MRI	5.36	±1.48	44.19	±7.08
Ultrasound	5.26	±1.51	43.49	±7.08
Mean difference	0.097	±0.22	0.7	±0.26

**Table 2:** Pathologies found in the ultrasound examination and arthro-MRI.

	N	%
Presence of an asphericity in the US	51.0	100.0
Presence of an asphericity in MRI	47.0	92.2
Labral tear in MRI	49.0	96.1
Cartilage lesion in MRI	25.0	49.0

## Data Availability

The data presented in this study are available on request from the corresponding author. The data are not publicly available due to privacy.
